# Estimation of Chlorophyll-a Concentration and the Trophic State of the Barra Bonita Hydroelectric Reservoir Using OLI/Landsat-8 Images

**DOI:** 10.3390/ijerph120910391

**Published:** 2015-08-26

**Authors:** Fernanda Sayuri Yoshino Watanabe, Enner Alcântara, Thanan Walesza Pequeno Rodrigues, Nilton Nobuhiro Imai, Cláudio Clemente Faria Barbosa, Luiz Henrique da Silva Rotta

**Affiliations:** 1 Department of Cartography, Sao Paulo State University, Cep. 19060-900, Presidente Prudente, SP 19060-900, Brazil; E-Mails: twalezsa@gmail.com (T.W.P.R.); nnimai@fct.unesp.br (N.N.I.); luizhrotta@yahoo.com.br (L.H.S.R.); 2 Image Processing Division, National Institute for Space Research, São José dos Campos, SP 12227-010, Brazil; E-Mail: claudio@dpi.inpe.br

**Keywords:** chlorophyll-*a*, bio-optical models, case-2 waters, remote sensing, multispectral image

## Abstract

Reservoirs are artificial environments built by humans, and the impacts of these environments are not completely known. Retention time and high nutrient availability in the water increases the eutrophic level. Eutrophication is directly correlated to primary productivity by phytoplankton. These organisms have an important role in the environment. However, high concentrations of determined species can lead to public health problems. Species of cyanobacteria produce toxins that in determined concentrations can cause serious diseases in the liver and nervous system, which could lead to death. Phytoplankton has photoactive pigments that can be used to identify these toxins. Thus, remote sensing data is a viable alternative for mapping these pigments, and consequently, the trophic. Chlorophyll-*a* (Chl-*a*) is present in all phytoplankton species. Therefore, the aim of this work was to evaluate the performance of images of the sensor Operational Land Imager (OLI) onboard the Landsat-8 satellite in determining Chl-*a* concentrations and estimating the trophic level in a tropical reservoir. Empirical models were fitted using data from two field surveys conducted in May and October 2014 (Austral Autumn and Austral Spring, respectively). Models were applied in a temporal series of OLI images from May 2013 to October 2014. The estimated Chl-*a* concentration was used to classify the trophic level from a trophic state index that adopted the concentration of this pigment-like parameter. The models of Chl-*a* concentration showed reasonable results, but their performance was likely impaired by the atmospheric correction. Consequently, the trophic level classification also did not obtain better results.

## 1. Introduction

The construction and use of hydroelectric dams and reservoirs change the hydrodynamics of rivers, causing diverse impacts to terrestrial and aquatic systems [1]. The increase in residence time leads to the availability of nutrients for a longer time and, consequently, to eutrophication [2]. Eutrophication is a natural process in aquatic systems, characterized by the over-enrichment of waters with nutrients, such as phosphorus and nitrogen [3,4].

However, this process can be accelerated by human inputs of nutrients [5], through the discharge of domestic, agricultural, and industrial effluents [6], known as cultural eutrophication [7]. Eutrophication increases primary productivity, represented by algae communities, such as phytoplankton [4,5]. Phytoplankton has an important role in aquatic systems. Furthermore, phytoplankton is responsible for the ocean being considered a carbon sink and an ally against global warming [8,9,10].

On the other hand, reservoirs have been appointed as potential emitters of greenhouse gases [11], although there are reservoirs with high phytoplankton concentrations [2,12]. Studies of Brazilian hydroelectric reservoirs showed that greenhouse gases were emitted by these aquatic systems due to high eutrophication levels [12,13,14,15]. Furthermore, some species present pigments that increase the efficiency of light and nitrogen absorption, such as cyanobacteria, favoring their predominance in different aquatic environments [16]. Some of these species produce toxic metabolites that can cause minor illnesses in the liver and nervous system [17,18,19,20]. Hence, the monitoring and classification of trophic levels are important for identifying the waters’ health.

Photosynthetically active pigments, such as chlorophylls and carotenoids, can be used as proxies for results these organisms. Chlorophyll-*a* (Chl-*a*) is the main pigment found (usually in larger amounts) in all phytoplankton species, and the Chl-*a* concentration is used for primary productivity studies and the determination of phytoplankton biomass. Due to a high correlation between the Chl-*a* and nutrient concentrations, this pigment was used as a proxy of trophic status. To determine the trophic status of rivers, lakes, and reservoirs, researchers created different ways of classifying them. Trophic State Index (TSI) [21,22,23] uses phosphorus concentration, Secchi disk transparency, and Chl-*a* concentration as parameters of the equation.

The sampling scheme based on punctual measurement collection is a time, money and human resources-consuming method that does not represent spatial variability. One way to overcome these limitations is by using remote sensing [24]. Remote sensing offers a significant source of information that can be used in methods for the operational large-scale monitoring of water quality [25]. Remote sensing has great potential to detect pigments, such as Chl-*a*, because their constituents are photo-active and their reflected energy can be measured by photosensitive sensors [26,27].

In the last decades, several bio-optical models were developed to estimate pigment concentrations and other optically active constituents (OAC) from hyperspectral (HICO, Hyperion) or moderated spectral resolution images (MERIS, MODIS and SeaWiFis) [28,29,30,31,32,33,34]. Previous studies [35,36,37,38] used multispectral images, such as Landsat, to estimate phytoplankton pigments. A trophic state classification can be applied using the estimated Chl-*a*. [39] evaluated the potential of a field spectrometer and IRS-1C satellite image to monitor the Chl-*a* content and trophic state of a lake in Germany. This lake belonged to a complex aquatic system and presented oligotrophic characteristics, with algal blooms during specific periods of the year.

The Operational Land Imager (OLI) sensor was launched onboard the Landsat-8 satellite in February 2013. The OLI sensor has shown the potential for applications in assessing water quality, and it is freely available. In [40] its authors conducted an analysis of the OLI’s radiometric performance in applications of the aquatic sciences, such as the retrieval of optically active constituents and benthic mapping. This study showed that OLI derived water-leaving radiance and reflectance presented some discrepancy when compared to *in situ* measurements or MODIS Aqua bands.

In [41] OLI images were used to study offshore suspended sediment concentrations. Their results showed that the OLI spatial resolution and signal noise ratio was suitable to study the offshore suspended sediment concentration. A study in the Amazon [42] used a time-series (from 1973 to 2013) of Landsat-MSS/TM/OLI images to monitor the impacts of mining actives near the Tapajós River (Amazon, Brazil) from the retrieval of the total suspended sediment (TSS). They obtained satisfactory results, showing that TSS concentration was in sync with the mining activities.

The main goal of this work was to map the seasonal trophic state of a tropical reservoir. Two approaches were used. The first approach was based on the Chl-*a* concentration retrieved from OLI/Landsat-8 images. The second approach was based on the Chl-*a* concentration and Secchi disk data collected during the two field surveys. The results of the second approach were considered as the ground truth. Both of the approaches used methods based on the classification adopted by the Environment Protection Agency in Sao Paulo State (CETESB) [43].

Two field campaigns were conducted to collect field spectroscopy data and water samples. These data were used to fit an empirical model to estimate the Chl-*a* concentration that was applied on time series of OLI/Landsat-8 images from May 2013 to October 2014. The classification of the trophic level was carried out for each OLI scene using the retrieved Chl-*a* data. The Chl-*a* retrieved in the laboratory and the Secchi disk transparency data collected *in situ* were used to classify the trophic level at each sampling spot. 

## 2. Study Area

**Figure 1:**
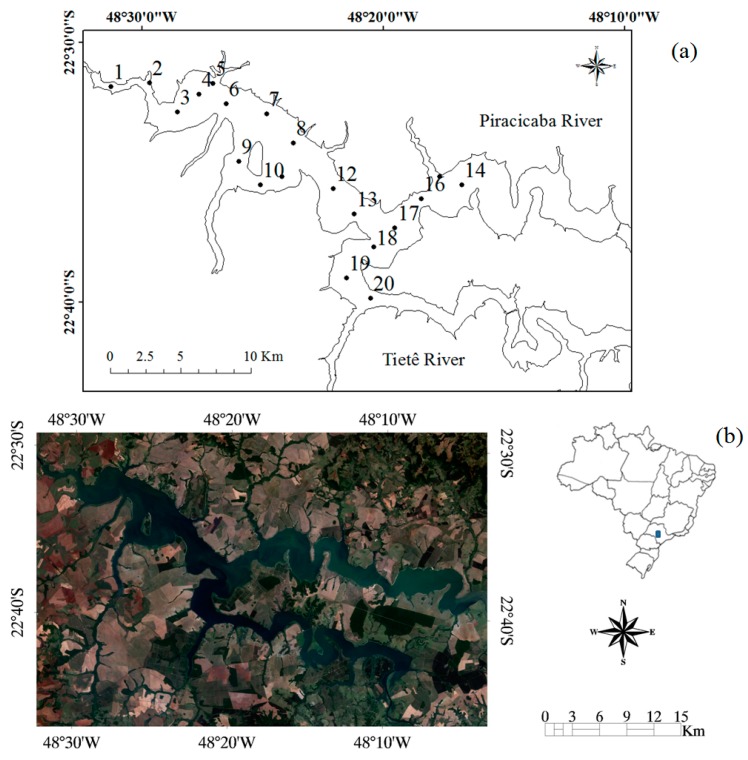
Study area-BBHR, Tietê River, São Paulo State. (**a**) Sampling spot locations; (**b**) OLI/Landsat-8 image of October 13, 2014, colored composition RGB-432, and Barra Bonita reservoir localization inside of Brazil.

**Figure 2:**
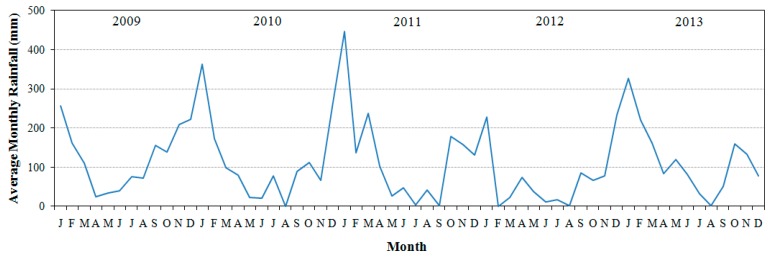
Plot of average monthly precipitation from January 2009 to December 2013 (5 years) associated to the Barra Bonita automatic station.

The study area was the Barra Bonita hydroelectric reservoir—BBHR (22°31′10″ S and 48° 32′3″ W), located in the middle course of the Tietê River, São Paulo State, Brazil (Figure 1). The BBHR is used not only for power generation but also for many other uses, such as recreation and tourism, fisheries, and aquaculture [44]. The BBHR is the first of six reservoirs cascading in the Tietê River. It was built in 1963, flooding an area of 310 km^2^ with a volume of 3.622 × 10^6^ m^3^. It is one of the two accumulation reservoirs in the dam chain and presents a considerable variation of quota from 439.5 m to 451.5 m [45]. The BBHR is located in a transitional region between tropical to subtropical [2], characterized by a dry period between May–October and a wet period between November and April [46] (Figure 2).

According to [45] and [44], the maximum depth is 25 m, with an average of 10.2 m, and the water retention time varies from 30 days (austral summer) to 180 days (austral winter). The flow range is over 1500 m^3^·s^−1^ in the summer (wet season) and approximately 200 m^3^·s^−1^ in the winter (dry season).

The BBHR is formed by the confluence of the Piracicaba and Tietê Rivers. The rivers are both located in highly populated and industrial areas, contributing high load of nutrients and pollution into the water. The BBHR is considered a eutrophic water body, mainly due to the discharge of wastewater and agricultural fertilizers [46]. In [12] it was shown that the eutrophication process caused greenhouse gas emissions, such as CH_4_ (methane), CO_2_ (carbon dioxide) and N_2_O (nitrous oxide) in the BBHR. These processes occurred due to the decomposition of the organic matter, releasing CH_4_ and CO_2_ [11], and denitrification, releasing NO and N_2_O to the atmosphere [12]. 

Historically, the BBHR presents a species richness and high concentration of phytoplankton [47,48]. Reference [47] investigated the phytoplankton species diversity in the reservoir during two years (1993–1994). The authors verified the predominance of the species *Microcystis aeruginosa* in summer, with high diversity in the winter. In [48] the authors conducted a taxonomic analysis during two years (2002–2004) and recorded nine groups. The most abundant species and *M. aeruginosa* and free cells of *Microcystis sp*. (Cyanophyceae), and *Aulacoseira granulata* filaments (Bacillariophyceae). The variation of phytoplankton communities was mainly associated with vertical mixing and residence time.

## 3. Data and Methods

### 3.1. Field Survey

Two field campaigns were conducted in distinct seasons of the year. The first field survey was accomplished from 5 to 9 May 2014 (Austral Autumn–end of the wet season). The second field survey was conducted from 13 to 16 October 2014 (Austral Spring–end of the dry season). The field survey dates were chosen to coincide with the Landsat satellite overpass in periods of low rainfalls and during the ends of the flood and drought periods. Unfortunately, there was high cloud cover during the satellite overpass for the survey in May.

Radiometric and limnological data, inherent optical properties (IOPs), and water samples were collected in 20 sampling spots (Figure 1). The sampling spots were randomly distributed along the reservoir. A semi-automatic approach was applied for selecting the points, adapted from [49]. First, the standard deviation was calculated from an annual image series composed of nine ETM+/Landsat 7 scenes (without clouds) from 17 August 2002 to 14 June 2003. A stratified random sampling was applied to the standard deviation using Hawth’s Analysis Tools (GME, Brisbane, Australia) for ArcGIS software (ESRI, Redlands, CA, USA). A 1 km buffer from margin was created to avoid optically shallow waters. The points within this buffer were discarded. This last step was especially important because the drought of that year considerably reduced the level of water.

#### Field Measurements

Radiometric data were measured using three RAMSES spectroradiometers. One spectroradiometer was an irradiance sensor (ACC-VIS), and the other two were radiance sensors (ARC-VIS) with a 7° field-of-view. ARC-VIS and ACC-VIS RAMSES work in a wavelength range of 320 nm to 950 nm and spectral sampling of 3.3 nm. The operation temperature varied from −10 °C to +50 °C, while the integration time range was 4 ms to 8 s [50]. The cosine collector was pointed upward and coupled to a 1.5 m rod for collecting the incident sky radiation, $E_{s}(\lambda )$ (W·m^−2^). Meanwhile, a radiance sensor was pointed in the upward direction of 135° to measure the incident sky radiance $L_{s}(\lambda )$ (W·m^−2^·sr^−1^). The second radiance sensor was pointed in the downward direction of 45° to measure the total radiance, $L_{t}(\lambda )$ (W·m^−2^·sr^−1^). Lt is the sum of the water-leaving radiance $L_{w}(\lambda )$ (W·m^−2^·sr^−1^) plus any $L_{s}(\lambda )$ (W·m^−2^·sr^−1^) that was reflected by the water surface in the sensor direction, $L_{r}(\lambda )$ (W·m^−2^·sr^−1^) [51]. All of the sensors simultaneously collect the measurements. The geometry of acquisition has considered [51], and [52]. These data were used to calculate the remote sensing reflectance, $R_{rs}$ (sr^−1^) [50]:

(1) $$R_{rs}=\frac{\left(L_{t}-\rho L_{s}\right)}{E_{s}}$$

where $L_{t}(\lambda )$ is the measurement obtained by the sensor pointed at the water surface; $L_{s}(\lambda )$ is the incident sky radiance; $E_{s}(\lambda )$ is the incident sky irradiance; and ρ is a reflectance factor related to direction, wavelength, wind speed, sensor FOV, and sky radiance distribution [51,52]. Furthermore, optical water quality parameters were collected *in situ*, such as turbidity and Secchi disk depth at each sampling station. In the first field survey, 19 radiometric samples were successfully collected, and in 20 radiometric samples were successfully collected in the second survey.

### 3.2. Laboratory Analysis

Water samples were collected at each sampling spot and filtered through a Whatman GF/F glass fiber filter, 47 mm diameter and 0.7 μm pore size, to estimate the Chl-*a* concentration in the laboratory. The filter was frozen and kept in the dark until further analysis. The Chl-*a* concentration was estimated using the extraction by acetone method [53].

Water samples were filtered through a Whatman GF/F glass fiber filter (47 mm diameter and 0.7μm pore size) and stored frozen and in the dark to estimate TSS. In this case, the filters used in the filtration were previously ignited at 550 °C for 30 min and weighed. In the laboratory, the filters were dried in an oven at 105 °C for 12 h, desiccated and weighed to obtain the TSS. After, the filters were ignited at 550 °C for 30 min in the muffle furnace, desiccated and weighed to acquire the inorganic suspended solids (ISS). Subtracting the ISS from the TSS obtained the organic suspended solids (OSS), and dividing each component of solids determined their concentrations [54].

The absorption coefficient of each AOC was determined by spectrophotometry. Water samples were filtered with Whatman GF/F glass fiber filters, kept frozen and in the dark until the analysis to estimate the absorption coefficient by phytoplankton, detritus and total particulate material. The measurements were acquired over the 280–800 nm spectral range in 1 nm by using a 2600 UV-Vis spectrophotometer (Shimadzu, Kyoto, Japan) with dual beam and integrating sphere. The optical density of the particulate material was obtained from the first reading. After, the pigment in the filter was extracted with sodium chloride and the filter was measured again to determine the optical density of the detritus. These optical densities were corrected for multiple scattering effects caused by the glass-fiber filter [55]. The particles and detritus absorption coefficients were estimated from the corrected optical densities [56]. The phytoplankton absorption coefficient was obtained by subtracting the detritus absorption coefficient from the particles absorption coefficient.

Water samples were filtered through a Whatman nylon membrane filter with 0.22 μm pore size and 47 mm diameter to measure the CDOM optical density. The filtrate water sample was stored, kept cool and in the dark until the analysis. Samples were measured at room temperature in a spectral range of 280–800 nm using a quartz cuvette with 10 cm optical path in a Shimadzu 2600 UV-Vis spectrophotometer with a single beam. Milli-Q water was used as blank reference. The CDOM absorption coefficients were calculated from Equation (2) [57]:

(2) $$a_{CDOM}=2.3\frac{A_{CDOM}(\lambda )}{r}$$

where $A_{CDOM}(\lambda )$ is the optical density at wavelength (λ) and r is the cuvette path length in meters. This value was corrected for particles backscattering, using 700 nm as the reference wavelength [57].

### 3.3. OLI Image Processing

The empirical models for estimating Chl-*a* concentration were fitted to be applied on multispectral images from OLI/Landsat-8, freely available from the United States Geological Survey [58]. Most of the OLI bands have a terrestrial application. However, the bands with applications on vegetation and photosynthetic pigments can be suitable for estimating phytoplankton pigment concentrations in highly productive water bodies. The advantages of using OLI/Landsat-8 images are the acquisition of data every 16 days and their free availability.

The study area was located in the path/row 220/76. In this work, available OLI/Landsat-8 images were used since its launch until October 2014. After a preliminary analysis of the images, only those without cloud cover were selected. Eight scenes from 19 May 2013 to 13 October 2014 were used. Dates of the scenes used in this work were: 19 May 2013; 4 June 2013; 23 August 2013; 8 September 2013; 13 December 2013; 30 January 2014; 11 September 2014; and 13 October 2014. The first day of the field survey overlapped with the overpass of the OLI sensor on 13 October 2014.

The radiometric calibration was conducted to convert digital numbers into top-of-atmosphere radiance, using the metadata released with the images. The retrieval of the at-surface reflectance was accomplished using the Fast Line-of-sight Atmospheric Analysis of Spectral Hypercubes (FLAASH), an atmospheric correction module, implemented in the ENVI software. This tool adopted the MODerate resolution atmospheric TRANsmission (MODTRAN4), an atmospheric radioactive transfer code [59]. Agricultural activities, such as sugar cane, orange, and coffee cultures, were the main activities in the region of the BBHR. Ethanol production is the main industrial activity. Therefore, the tropical atmospheric model and rural aerosol model were selected for correction.

The cirrus band was included in the atmospheric process. The cirrus band was used to detect clouds and water vapor contents that were not detectable in the other bands [58]. Atmospheric aerosols can be liquid or solid particles suspended in a gas [60]. Rayleigh atmospheric scattering (aerosol scattering) affects the direction of short wavelengths, resulting in haze in the blue and green bands [61]. The inclusion of the cirrus band helped to eliminate the estimation of negative at-surface reflectance by FLAASH at short wavelengths (blue region). Negative values were associated with the overestimation of aerosol reflectance [62,63,64].

The surface reflectance values were divided by π [65,66] to convert them into *R_rs_*. Five samples (see P1, P2, P4, P5, and P6 in Figure 1) were collected at the time of the Landsat-8 overpass (13 October 2014) and were used to evaluate the performance of the atmospheric correction. Optical closure was conducted between the *R_rs_* obtained of the image and the simulated OLI *R_rs_* figured out from the field spectroscopy data. 

### 3.4. Model Calibration

Only two-band models were tested due to the limited number of OLI/Landsat-8 bands that could be used to estimate Chl-*a*. The structure proposed by [67] was adopted for fitting an empirical model for the retrieval Chl-*a* concentrations using OLI/Landsat-8 bands and *in situ* data (Equation (3)):

(3) $$Chl-a\;\text{concentration}\propto R_{rs}^{-1}(\lambda_{1})\times R_{rs}(\lambda_{2})$$

In this work, empirical models were fitted from the simulated OLI bands. The *R_rs_* for OLI bands were simulated (*R_rsL8k_*) using OLI’s spectral response [68], from the field radiometric data set composed of 39 measurements collected in the two field campaigns, as Equation (4):

(4) $$R_{rsL8k}=\frac{\int_{\lambda_{1k}}^{\lambda_{2k}}L_{w}(\lambda )\;S_{k}(\lambda )\;d\lambda }{\int_{\lambda_{1k}}^{\lambda_{2k}}E_{d}(\lambda )\;S_{k}(\lambda )\;d\lambda }$$

where *S_k_* (λ) is the radiometric sensitivity of band *k* that extends from lower wavelength *λ_1k_* up to upper wavelength *λ_2k_*. SWIR-1 and SWIR-2 bands were not simulated because their wavelengths exceeded the spectral range collected by the RAMSES spectroradiometer [50].

The proposed of this work to fit a unique model that could be applied to time series of OLI/Landsat-8 images of the BBHR. Therefore, samples gathered from two field campaigns (May and October) were used to calibrate the empirical model. The five samples collected at the time of the satellite overpass (13 October 2014) were used for validation. From the other 34 samples, it was done selection of the calibration samples. Samples must be within the prediction interval, considering a significance level of 0.95. Twenty-six samples were used to calibrate the empirical bio-optical model. 

Parameterization was conducted using the Interactive Correlation Environment (ICE) [69]. ICE is a statistical web tool for data collinearity analysis that determines the correlation of a dependent variable over the ratio between two independent variables. In this case, the dependent variable was the Chl-*a* concentration and the independent variables were OLI/Landsat-8 bands. The bands ratio (*R_rs_*) that was more correlated with the Chl-*a* concentration was used to develop a simple two-band model. The models were fit using the least-squared method and the data set composed of 26 samples. Linear, polynomial, and exponential fits were tested.

### 3.5. Tuning Models and Application of Existing Models

Other bands algorithms proposed by different researchers were tested. The bands selection of these algorithms were based on different sensors with specific bands for water, such as MERIS and MODIS, and made it possible to test models with more than two bands. First, band combinations proposed by different authors were tuned from the calibration using the spectroscopy data collected in the two field campaigns. The models tested were the two-band and three-band algorithms developed by [67], originally for terrestrial application, with bands selected by Moses *et al.* [70] and the Normalized Difference Chlorophyll Index (NDCI) developed by [32]. The applied band combinations, references, and adopted abbreviations are shown in Table 1.

**Table 1:** Structure and bands adopted by two-band and three-band algorithms, and NDCI.

Abbreviation	Bands Combination	Reference
2B	$R_{rs}(708)\times \;R_{rs}{\left(665\right)}^{-1}$	[70]
3B	$\left(R_{rs}{\left(665\right)}^{-1}\;-\;R_{rs}{\left(708\right)}^{-1})\;\times \;R_{rs}(753\right)$	[70]
NDCI	$\left(R_{rs}(665)\;-R_{rs}(708)\right)/(R_{rs}(665)\;+\;R_{rs}(708))$	[32]

The calibrations proposed in the literature [32,70,71,72] were also tested. MERIS bands were used to compose the two-band and three-bands algorithms. Table 2 shows the coefficients obtained by different researchers. The researchers proposed linear, polynomial of second degree, and exponential fits. These models were calibrated using a large dataset of different seasons and aquatic environments.

**Table 2:** Coefficients of calibration proposed in the literature using 2B and 3B and NDCI.

Reference	Index	a *	b *	c *	d *
[70]	2B	−37.94	61.324	−	−
	3B	23.174	232.29	−	−
[71]	2B	−19.3	35.75	−	1.124
	3B	16.45	113.36	−	1.124
[72]	2B	−15.18	14.85	25.28	−
	3B	25.66	215.95	315.5	−
[32]	NDCI	14.039	86.115	194.325	−

* Linear fit y = a + bx; polynomial fit y = a + bx + cx^2^; Exponential y = (a + bx)^d^.

### 3.6. Validation

The algorithms performance was evaluated using the following statistic metrics: normalized root mean squared error (NRMSE) (Equation (5)); the RMSE (root mean squared error) (Equation (6)); mean absolute percentage error (MAPE) (Equation (7)); bias (Equation (8)); mean normalized bias (MNB) (Equation (9)); normalized root mean squared error (NRMS) (Equation (10)); and determination coefficient (R^2^):

(5) $$NRMSE=\frac{RMSE}{\left(x_{maximo}^{measured}-x_{minimo}^{measured}\right)}$$

(6) $$RMSE=\sqrt{\frac{1}{n}\sum_{i=1}^{n}{\left(x_{i}^{estimated}-x_{i}^{measured}\right)}^{2}}$$

(7) $$MAPE=\frac{\sum_{i=1}^{n}\left(\left|\frac{x_{i}^{estimated}-x_{i}^{measured}}{x_{i}^{measured}}\right|\right)}{n}$$

(8) $$bias=\frac{1}{n}\sum_{i=1}^{n}\left(x_{i}^{estimated}-x_{i}^{measured}\right)$$

(9) $$MNB=\text{mean}\;(\varepsilon_{i})\;\%$$

(10) $$NRMS=\text{stdev}\;(\varepsilon_{i})\;\%$$

where $\varepsilon_{i}$ is the systematic and random error (Equation (11)):

(11) $$\varepsilon_{i}=100\times \frac{\left(x_{i}^{estimated}-x_{i}^{measured}\right)}{x_{i}^{measured}}$$

### 3.7. Trophic State Classification

The trophic state classification approach used was an adaptation from the method proposed by [18], conducted for CETESB [23,43], whose Equations (12), (13) and (14) correspond to the calculation from the Secchi disk transparency (*S*, m), Chl-*a* concentration (Chl*-a*, mg·m^−3^) and total phosphorus (*Pt*, mg·L^−1^), respectively:

(12) TSI(S) = 10×{6 − [(ln S)/ln 2]}

(13) TSI(Chla) = 10×{6−[(0.92−0.34×(ln Chla))/ln 2]}

(14) TSI(Pt) = 10×{6−[1.77−0.42×(ln Pt)/ln 2]}

Each TSI was separated into six tropic classes associated to a TSI range. Table 3 shows the classification of the reservoir’s trophic levels based on the TSI parameters and equivalent to total phosphorus, Chl-*a*, and Secchi disk transparency proposed by [23].

**Table 3:** Trophic state classification adopted by CETESB according to TSI and equivalence to the total phosphorus concentration (mg·L^−1^), Chl-a concentration (mg·m^−3^), and Secchi disk transparency (m) parameters.

Trophic State	Total Phosphorus (mg·L^−1^)	Chl-*a* (mg m^−3^)	Secchi Disk Transparency (m)	TSI
Ultraoligotrophic	Pt ≤ 0.008	Chl-*a* ≤ 1.17	S ≥ 2.4	TSI ≤ 47
Oligotrophic	0.008–0.019	1.17–3.24	2.4–1.7	47–52
Mesotrophic	0.019–0.052	3.24–11.03	1.7–1.1	52–59
Eutrophic	0.052–0.12	11.03–30.55	1.1–0.8	59–63
Supertrophic	0.12–0.2333	30.55–69.05	0.8–0.6	63–67
Hypertrophic	0.233 < Pt	69.05 < Chl-*a*	0.6 > S	TSI > 67

Reference [21] suggested that the choice of the indicator of the trophic state depends on the lake and seasonal period. According to [21], the use of Secchi disk transparency is not recommended for lakes with high nonalgal particles concentration, in highly colored lakes, and in extremely clear lakes. The main advantages of using a Secchi disk is the operational simplicity and low cost, and it usually has similar TSI values to Chl-*a*.

Reference [21] also suggested using the Chl-*a* concentration as the trophic state indicator because its values had less interference from other environment parameters. On the other hand, the accurate total phosphorus concentration as an indicator depends on it being the limiting factor for algal growth and being correlated to the algal biomass. Thus, the authors suggested classifying using a biological parameter, such as Chl-*a* in summer and total phosphorus in spring and fall. In this work, the classification was conducted using the Chl-*a* parameter and applied on all seasons.

## 4. Results

**Figure 3:**
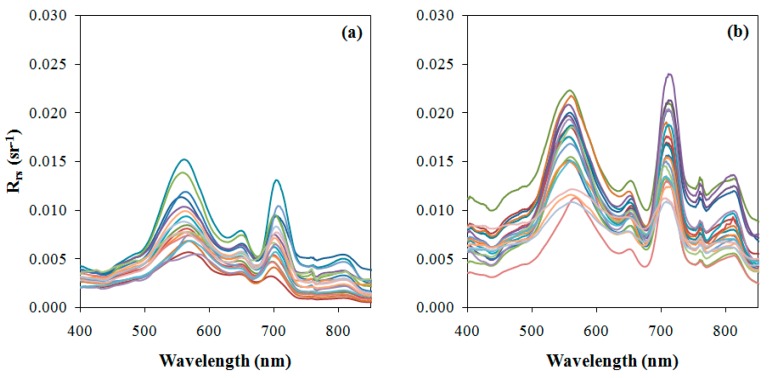
*R_rs_* spectra related to the field surveys conducted in (**a**) 5–9 May 2014, and (**b**) 13–16 October 2014.

Figure 3 shows *Rrs* spectra computed from *in situ* data collected on May 2014 (Figure 3a) and October 2014 (Figure 3b). The *R_rs_* curves presented characteristics of productive waters both in May and October. The Chl-*a* features were quite accentuated even in the spectra with lower Chl-*a* concentration (19.1 mg·m^−3^). All of the curves showed high absorption at the blue and red spectral regions, highlighting the reflection peak at the green region. Another feature associated with the presence of Chl-*a* was the peak at the beginning of near infrared (NIR) (region associated to red edge), at approximately 710 nm to 720 nm. Due to the high Chl-*a* concentration observed in October, the peak at NIR nm is highlighted in relation to the visible spectrum, and shifted slightly to the right. Another reflection feature was observed at 810 nm associated with both chlorophyll and organic matter [73].

In addition to Chl-*a* features, every *Rrs* spectrum presented an accentuated absorption feature at approximately 620 nm to 630 nm associated with the presence of phycocyanin, a pigment present in cyanobacteria [16,74]. The reflectance peak at approximately 650 nm to 660 nm was associated with phycocyanin fluorescence [16]. Phycocyanin has the characteristics of low absorption in blue and green regions [16]. Historically, *Microcystis* was the predominant species in the BBRH [47,48], which explained the phycocyanin and Chl-*a* spectral features. Moreover, high phycocyanin concentrations are a concern from the environmental and public health point of view. The BBHR has multiple uses of direct and indirect contact, such as recreation and fish intake. 

There was an increase considerable of the high phytoplankton, CDOM, and particles absorption coefficients from May to October. The average particles absorption coefficients were 1.6 m^−1^ in May and 2.8 m^−1^ in October (Table 4). The average Chl-*a* concentration was 122.5 mg·m^−3^ in May, while in October the average was 428.7 mg·m^−3^ (Table 4). The Chl-*a* concentrations ranged from 19.1 mg·m^−3^ to 293.3 mg·m^−3^ in May, and from 263.2 mg·m^−3^ to 797.8 mg·m^−3^ in October. It is important to note that the minimum Chl-*a* concentration measured in October (263.2 mg·m^−3^) was near the maximum concentration collected in May (293.2 mg·m^−3^).

**Table 4:** Descriptive statistics of the optical water quality parameters measured *in situ* or determined in the laboratory: Chl-*a* concentration; Secchi disk depth; turbidity; TSS concentration; OSS concentration; ISS concentration; OSS/TSS ratio; ISS/TSS ratio; phytoplankton absorption coefficient at 665nm; CDOM absorption coefficient at 440nm; total particulate material absorption coefficient at 440nm; and wind speed. Statistics metrics used: minimum value (Min), maximum value (Max), median, median, standard deviation (SD) and coefficient of variation (*CV*) in percentage (%), that is *CV* = (SD / mean) × 100.

**a. Dataset measured on 5–9 May 2014 at 19 stations**						
**Parameter**	**Min**	**Max**	**Mean**	**Median**	**SD**	***CV* (%)**
Chl-*a*, mg m^−3^	19.1	293.2	122.5	105.9	72.3	60
Secchi disk depth, m	0.8	2.3	1.5	1.4	0.4	30
Turbidity, NTU	1.7	12.5	5.2	5	2.5	50
TSS, mg L^−1^	3.6	16.3	7.0	6.5	3.2	50
OSS, mg L^−1^	2.8	14.7	5.9	4.8	3.1	50
ISS, mg L^−1^	0.2	4.4	1.1	0.8	0.9	80
OSS/TSS	0.45	0.98	0.83	86	11.6	10
ISS/TSS	0.02	0.55	0.17	14	11.6	70
*a_phy_* (665), m^−1^	0.2	1.3	0.6	0.5	0.3	50
*a_CDOM_* (440), m^−1^	0.6	1.1	0.8	0.8	0.1	10
*a_p_* (440), m^−1^	0.7	2.9	1.6	1.5	0.7	40
Wind, m s^−1^	0.6	4.9	1.8	1.6	1.1	60
						
**b. Dataset measured on 13–16 October 2014 at 20 stations**						
**Parameter**	**Min**	**Max**	**Mean**	**Median**	**SD**	***CV*(%)**
Chl-*a*, mg m^−3^	263.2	797.8	428.7	368.9	154.5	40
Secchi disk depth, m	0.4	0.8	0.6	0.6	0.1	20
Turbidity, NTU	11.6	33.2	18.6	17.6	5.3	30
TSS, mg L^−1^	10.8	44	22	21.2	7	30
OSS, mg L^−1^	10.2	30.4	18.2	18.4	4.8	30
ISS, mg L^−1^	0.6	3.8	2.6	2.8	1	40
OSS/TSS	0.8	1	0.9	0.9	0.1	10
ISS/TSS	0.04	0.2	0.1	0.1	0.4	10
*a_phy_* (665), m^−1^	0.6	2.1	1.1	1	0.4	40
*a_CDOM_* (440), m^−1^	0.9	2.4	1.3	1.3	0.3	30
*a_p_* (440), m^−1^	1.6	5.6	2.8	2.6	1	40
Wind, m s^−1^	0	5	1.5	1.1	1.5	100

The TSS concentration was mainly composed of organic particles (OSS) (approximately 90%), indicating that TSS could be directly related to phytoplankton. Nevertheless, the complexity of the BBHR must not be neglected during the development of the models. An ISS of approximately 4 mg·L^−1^ can mask some features of pigments, mainly in the visible region.

As expected, increasing turbidity influenced the Secchi disk transparency. Maximum turbidity was 12.5 NTU (Nephelometric Turbidity Unit) in May and 33.2 NTU in October. The Secchi disk depth range was 0.8 m to 2.3 m in the first field campaign, while the Secchi disk depth did not exceed 0.8 m in the second field campaign. Furthermore, the elevation of the TSS and pigment concentration reflected the particles absorption coefficient (*a_p_*) and phytoplankton absorption coefficient (*a_phy_*), respectively.

The collection of radiometric measurements was not affected by the influence of waves in both field campaigns. Except for a few sampling spots, the wind speed was close to 0 m·s^−1^.

**Figure 4:**
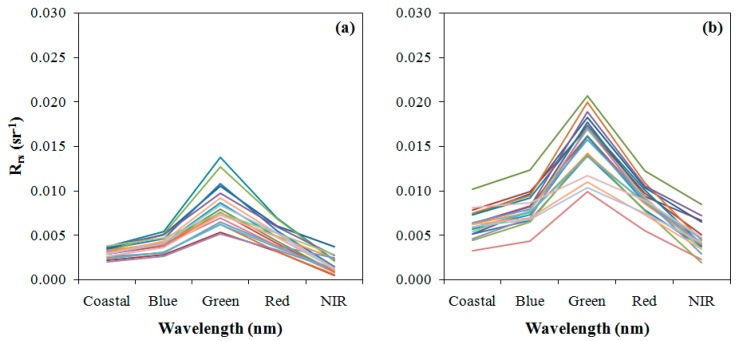
*R_rs_* simulated for OLI/Landsat-8 bands of the spectra collected in (**a**) May 2014 and (**b**) October 2014.

Simulation of the OLI bands was accomplished using the spectra plotted in Figure 3. Figure 4 shows the simulated Rrs curves. As expected, the features associated with the absorption by phycocyanin (approximately 630 nm) and reflectance by chlorophyll-a (near 720nm) disappeared. The high absorption at the green region and low absorption at the blue region, associated to chlorophyll-a presence, were maintained. Simulated bands were used to select and calibrate the empirical models.

**Figure 5:**
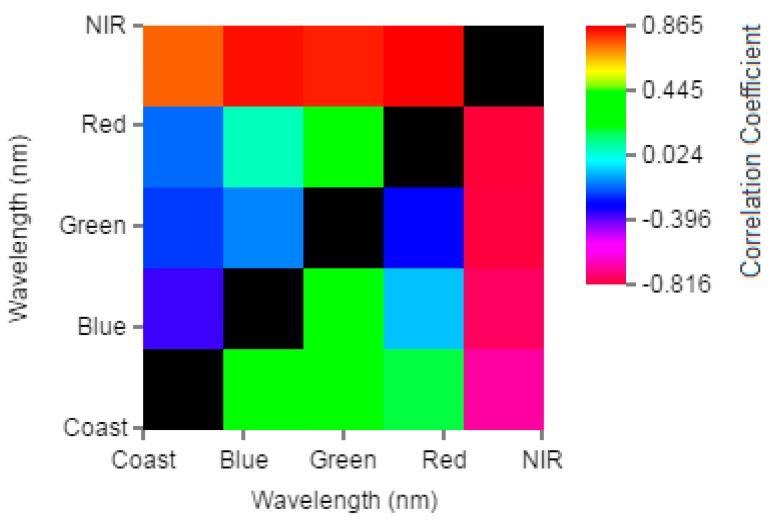
2-Dimensional plot of the correlation coefficients (R) between bands ratio and Chl-*a* concentration.

The bands were selected using the web tool ICE, and the result is presented in Figure 5. The 2-Dimensional correlogram shows the correlation between the bands ratio and Chl-*a* concentration. As expected, the NIR-Red ratio showed the highest positive correlation (0.865) with Chl-*a*. The Red-NIR ratio presented the highest negative correlation of -0.8161. The NIR-Green and NIR-Blue also showed good correlation (greater than 0.8).

**Figure 6:**
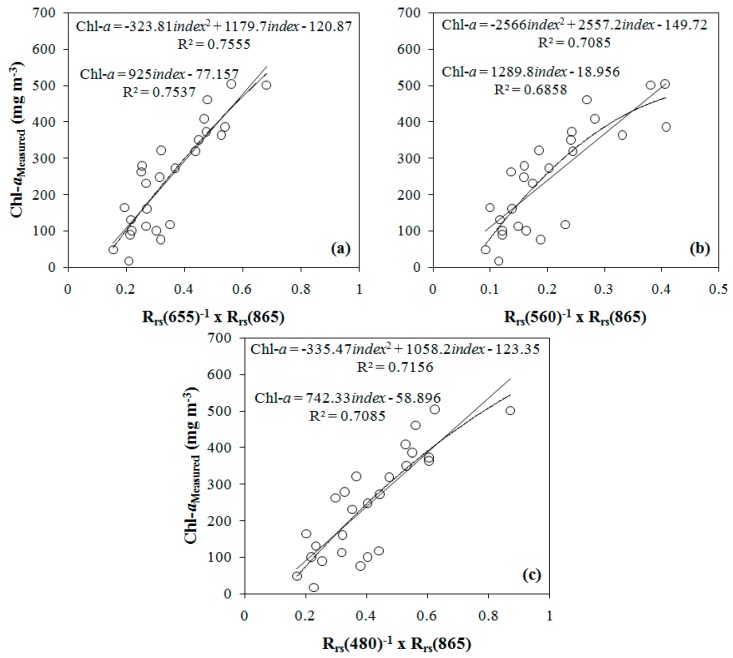
Two-band models developed from the OLI bands simulated using field radiometric data, using (**a**) NIR-Red ratio, (**b**) NIR-Green ratio, and (**c**) NIR-Blue ratio.

These bands were tested in the development of an empirical model. The models calibration was accomplished by a least-squared regression. Linear and polynomial fits were tested, obtaining satisfactory results (Figure 6). The adjustment line of regression and R^2^ showed that there were little differences between the linear and polynomial fits (Figure 6a). The NIR-Red ratio had the best calibrations, with R^2^ of 0.7537 to linear fit and R^2^ of 0.7555 to polynomial calibration. The NIR-Green (Figure 6b) and NIR-Blue (Figure 6c) ratios also presented satisfactory fits. The NIR-Blue ratio obtained R^2^ lower than 0.70, except for the linear fit. 

Table 5 shows the calibration parameters, determination coefficient of fit, and p-value statistics for each calibration using OLI/Landsat-8 bands. Table 5 shows the same parameters for tuned models using MERIS wavelengths proposed by different authors (Table 2). Among all of the calibrations tested, the best fits were obtained by the NIR-Red ratio using OLI/Landsat-8 bands. Among the models using MERIS wavelengths, the model 3B showed the best fit both linear and polynomial. The *p*-value statistics of the regressions were equal to zero or negligible; that is, the coefficients of the regression models were suitable to explain the Chl-*a* concentration.

**Table 5:** Fit parameters of the two-band models obtained from the simulated bands of the OLI/Landsat-8 sensor and bands algorithms tuned for bands of the MERIS and MODIS sensors (2B; 3B and NDCI): equations coefficients (a, b and c), R^2^ (determination coefficient), and *p*-value statistic.

Index	a	b	c	R^2^	*p*-value
NIR/Red (Linear)	−77.16	925.001	−	0.7537	0.00000
NIR/Green (Linear)	−18.96	1289.84	−	0.6858	0.00000
NIR/Blue (Linear)	−58.90	742.33	−	0.7085	0.00000
NIR/Red (Polynomial)	−120.87	1179.72	−323.81	0.7555	0.00000
NIR/Green (Polynomial)	−149.72	2557.24	−2565.99	0.7085	0.00000
NIR/Blue (Polynomial)	−123.35	1058.23	−335.47	0.7156	0.00000
2B (Linear)	−124.72	213.73	−	0.3910	0.0006
3B (Linear)	71.212	603.15	−	0.5794	0.00001
NDCI (Linear)	53.361	767.2	-	0.3945	0.0006
2B (Polynomial)	−214.41	321.62	−30.63	0.3926	0.0006
3B (Polynomial)	33.95	918.36	−465.68	0.5963	0.00001
NDCI (Polynomial)	56.818	724.88	93.472	0.3946	0.0006

Linear fit y = a + bx; polynomial fit y = a + bx + cx^2^

Table 6 shows the validation results of the fitted models from simulated OLI bands (Table 6a), the tuned models from the bands combinations proposed by literature (Table 6b), and the same bands combinations using calibrations proposed by different authors [31,64] (Table 6c). Among the models for OLI data (Table 6a), the polynomial NIR-Green model presented the best results in the validation. This model presented a NRMSE of 82.39%; MAPE of 36.02%; bias of -126.47; MNB of 35.13%; and R^2^ of 0.1929. The polynomial NIR-Blue model presented the second best results with a NRMSE of 86.54%; MAPE of 45.69%; bias of -137.90; MNB of 43.28%; NRMS of 31.47%; and R^2^ of 0.3441. Overall, the models developed using simulated OLI bands did not have satisfactory performances.

Tuned models yielded better results than the fitted models using simulated OLI/Landsat-8 data (Table 6b). Among the tuned models, polynomial and linear 3B models had the best performances with NRMSE of 16.71% and 18.70%, respectively. On the other hand, the NDCI model yielded the worst estimation, with MNB of 83.8%. However, the tuned NDCI performance was still better than the fitted models. Performances of the tuned 2B models were next to the tuned NDCI models.

Among the models proposed by the literature, the 3B model proposed by [72] showed the best performance, with NRMSE of 80.45% and MAPE of 43.57%. The 2B model proposed by [72] had the best results when compared to other 2B models tested (Table 6c). The NDCI calibration proposed by [32] presented the lowest performance, with NRMSE of 157.99% and MAPE of 81.72%.

**Figure 7:**
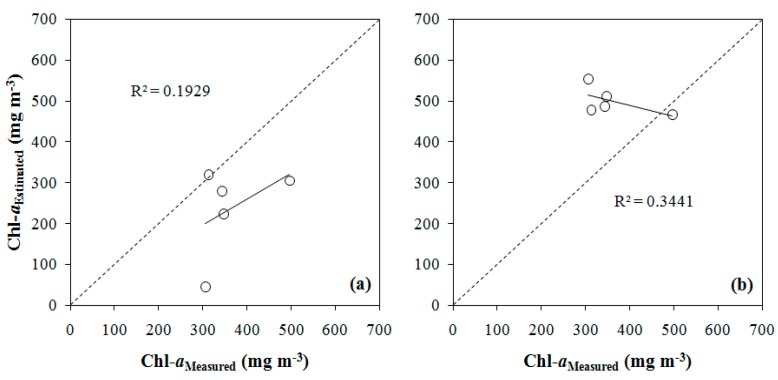
Two-band models validation, using (**a**) polynomial NIR-Green ratio and (**b**) polynomial NIR-Blue ratio.

Figure 7 shows plots 1:1 between the validation samples and Chl-*a* concentration estimated from the models using simulated OLI/Landsat-8 bands that had the best results in the validation, *i.e.*, NIR-Green and NIR-Blue models with polynomial fit. Overall, the polynomial NIR-Green model was the best model, with a satisfactory fit and the best results in the validation when compared to other fitted models using simulated OLI/Landsat-8 bands. Therefore, the bio-optical model was applied to the temporal series of OLI images from May 2013 to October 2014.

**Table 6:** Validation of bio-optical models applied for estimating Chl-*a* concentration from the NRMSE, MAPE, bias, MNB, NRMS, and R^2^.

**a. Models calibrated using ICE** [62] **from OLI bands simulated and data collected in the study area.**						
**Model**	**NRMSE** **(%)**	**MAPE** **(%)**	**Bias**	**MNB (%)**	**NRMS (%)**	**R^2^**
NIR/Red (Linear)	170.01	91.96	307.56	91.96	44.17	0.1921
NIR/Green (Linear)	190.91	103.90	348.61	103.90	47.00	0.2823
NIR/Blue (Linear)	100.78	52.32	163.16	50.84	36.12	0.3301
NIR/Red (Polynomial)	144.17	77.92	259.62	77.92	37.71	0.1934
NIR/Green (Polynomial)	**82.39**	**36.02**	**−126.47**	**−35.13**	32.38	0.1929
NIR/Blue (Polynomial)	86.54	45.69	137.90	43.28	**31.47**	**0.3441**
**b. Tuning bands combinations proposed by literature.**						
**Model**	**NRMSE** **(%)**	**MAPE** **(%)**	**Bias**	**MNB** **(%)**	**NRMS** **(%)**	**R^2^**
2B (Linear)	32.09	13.60	−51.71	−13.60	**8.39**	0.816
3B (Linear)	18.70	8.90	**0.75**	−0.47	11.62	0.7953
NDCI (Linear)	33.84	12.48	−49.56	−12.44	9.59	**0.867**
2B (Polynomial)	32.49	12.96	−50.26	−12.96	8.86	0.7918
3B (Polynomial)	**16.72**	**7.67**	−3.19	**−0.34**	9.69	0.7724
NDCI (Polynomial)	33.57	12.48	−49.54	−12.48	9.47	0.7759
**c. Models proposed by authors, calibrated using data from other environments**						
**Model**	**NRMSE** **(%)**	**MAPE** **(%)**	**Bias**	**MNB** **(%)**	**NRMS** **(%)**	**R^2^**
2B [70]	146.82	75.82	−274.69	−75.82	2.35	0.816
3B [70]	120.13	62.88	−226.17	−62.88	4.56	0.867
2B [71]	146.19	75.64	−273.87	−75.64	2.37	0.821
3B [71]	127.51	66.81	−240.23	−66.81	4.35	0.8723
2B [72]	128.11	66.68	−240.63	−66.68	3.84	0.841
3B [72]	**80.45**	**43.57**	**−151.14**	**−43.57**	13.62	**0.8975**
NDCI [32]	157.99	81.72	−295.85	−81.72	**1.85**	0.7978

**Figure 8:**
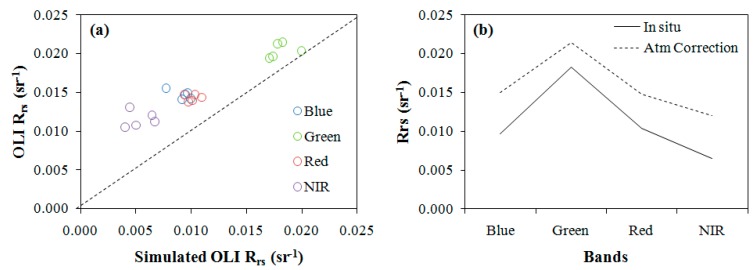
Validation of atmospheric correction. Optical closure of (**a**) FLAASH satellite *R_rs_ versus* simulated *R_rs_* for OLI data at Blue, Green, Red, and NIR bands and (**b**) comparison of *R_rs_* calculated for OLI imagery (dotted line) with simulated OLI *R_rs_* (solid line) at the time of the overpass.

An atmospheric correction was applied to OLI images so the model could be applied. Figure 8 shows the validation of the atmospheric correction. Plotting the OLI imagery *R_rs_ versus* simulated OLI *R_rs_* showed that *R_rs_* was overestimated (Figure 8a). Simulated OLI *Rrs* and OLI imagery *Rrs* spectra of the sampling point P1 are shown in Figure 8b. There is a difference in the magnitude between the *R_rs_* curves, but the shapes of both are very similar. Likely, difference in the magnitude was associated to the glint effect or aerosols that were not completely removed by the atmospheric inversion model. In models of only one band, the magnitude difference could underestimate or overestimate the estimations. However, the bands ratios eliminated this problem because the shape remained equal.

**Figure 9:**
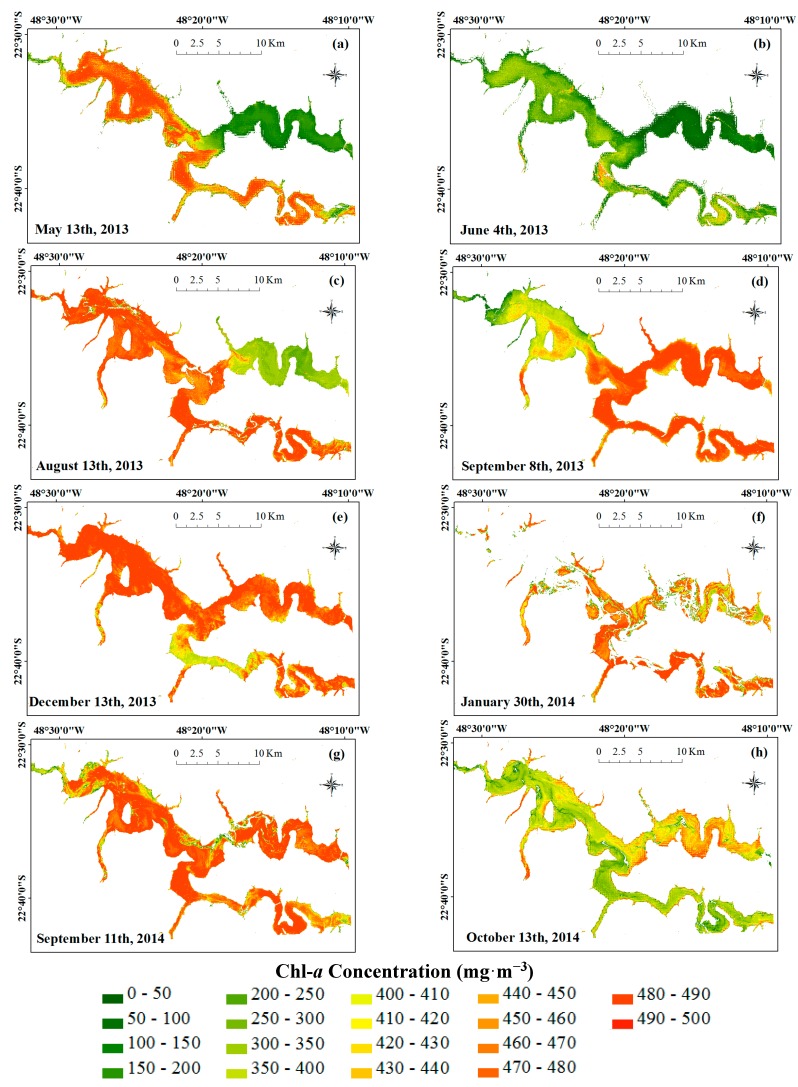
Maps of Chl-*a* concentration (mg·m^−3^) retrieved from OLI/Landsat-8 based on NIR-Green algorithm for (**a**) May 2013, (**b**) June 2013, (**c**) August 2013, (**d**) September 2013, (**e**) December 2013, (**f**) January 2014, (**g**) September 2014, and (**h**) October 2014.

Figure 9 shows the maps of the Chl-*a* concentration estimated by the empirical model using the NIR-Green ratio. Many pixels were estimated with negative values, showing that the model greatly underestimated the Chl-*a* concentration. These pixels were not represented in the maps (Figure 9a–h), but it can clearly see in Figure 9f (30 January 2014 image). January had extremely high Chl-*a* concentrations when compared to the other months, and the model was not able to suitably estimate high concentrations. The model yielded negative values in other months as well. Despite the model having underestimated high Chl-*a* concentration, variations of concentration were consistently observed throughout the reservoir. When the points in the map were compared to the field data, it verified that there was coherence in the areas of major and minor concentrations of Chl-*a*.

**Figure 10:**
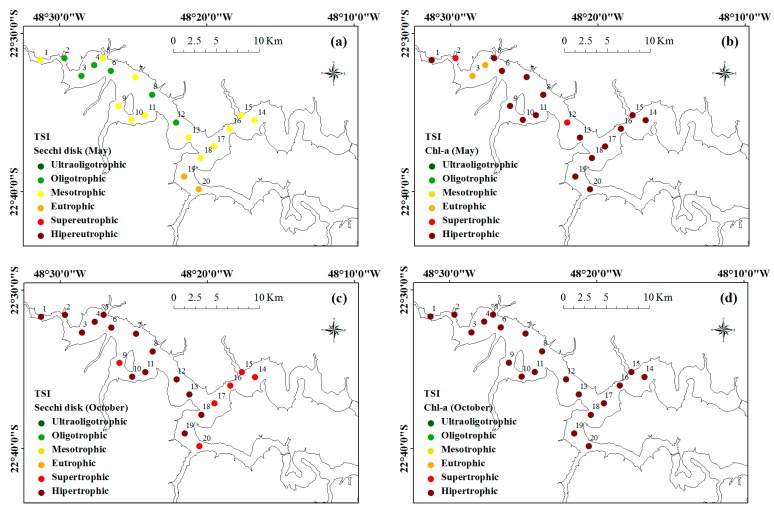
Trophic state classification: from Secchi disk transparency and Chl-*a* concentration. Classification for May according to (**a**) Secchi and (**b**) Chl-*a*, and October (**c**) Secchi and (**d**) Chl-*a*.

Figure 10 shows maps representing the points of the trophic state for May (Figure 10a,b) and October (Figure 10c,d) 2014. Each point represents the trophic level of a sample of Secchi disk transparency (Figure 10a,c) and Chl-*a* concentration (Figure 10b,d) collected in the field. It was clear that Secchi disk parameter tended to classify the water for lower trophic levels when compared to classification from Chl-*a* in both periods.

This difference was more visible in May than in October, when the trophic levels were lower. If there really was an underestimation, then sanitation issues may be of concern. In this situation, if the classification was conducted by Secchi disk, it would be regarded as the suitable for multiple uses. Nevertheless, this could be a mistake that could endanger public health.

Despite the classification from the Secchi disk in May that indicated an environment with lower trophic levels, other classifications showed the opposite. The predominance of hypereutrophic points from the classification by Chl-*a* was observed in both May and October. Reference [21] suggested that these parameters yielded similar trophic states in conditions of non-clear waters and the predominance of algal particles. Although algal particles were predominant in the BBHR, the classification using the Secchi disk and Chl-*a* yielded quite different results.

## 5. Discussion

Chl-*a* measurements collected *in situ* corroborated with the classifications of water quality that were classified by CETESB. From the beginning of monitoring, water quality was considered bad due to high levels of eutrophication. Chl-*a* concentrations were very high in May and October. Chl-*a* concentrations were far above the minimum value of hypertrophic waters, which has been determined as 69.05mg·m^−3^ [23,43]. This situation worsened due to the historic elevated proliferation and predominance of *M. aeruginosa* and *Microcystis sp*, species of cyanobacteria known to produce hepatoxin [47,48]. The BBHR is used for activities such as recreation and fishing. Direct contact with water or ingestion of fish can cause problems, from minor skin problems to microcystin toxin contamination. 

The fitted model in this work was able to represent the seasonal dynamic in the BBHR when there was no scum. Nevertheless, this model cannot accurately estimate the Chl-*a* concentration and, consequently, the trophic state. Analyzing Figure 9a–h, the lacustrine zone (the wide area before the river narrowing and next to the dam) [44] of the BBHR had high Chl-*a* concentrations when compared to other points of the reservoir, mainly in the Austral Spring and Austral Summer. Increasing residence time favored the development of algae in this region of the BBHR. The Tietê River, before the confluence with the Piracicaba River, also had high Chl-*a* concentrations when compared to other points of the reservoir. In this section of the BBHR, the eutrophication caused by a high discharge of pollutions coming from the metropolitan area of São Paulo city was the main reason for the proliferation of algae [2,12,44,45,75].

There were lower Chl-*a* concentrations in the Piracicaba River during periods of lower temperature, such May, June, and August (Austral Autumn and Winter) than in the other regions of the reservoir. This behavior was confirmed by the reports of Surface Water Quality in São Paulo State [43]. According to the data of three CETESB monitoring spots (located in Piracicaba River, Tietê River and the BBHR), the Piracicaba River was less eutrophic, while the Tietê River was more eutrophic [43]. In the Austral Spring the situation was different, and it was possible to identify the maximum concentration in some regions. However, at the beginning of the rainy season (Austral Summer), the dilution decreased the Chl-*a* concentration in this region again.

The trophic state classification (Figure 10) showed that the classification by the Chl-*a* parameter was more rigorous than Secchi disk parameter. In May, before the confluence, the Piracicaba and Tietê Rivers had higher trophic levels that decreased along the path. Two points approaching the dams were considered mesotrophic. However, as the channel narrows, the trophic level starts to rise. In the nearest point nearest to the dam, its classification was already hypereutrophic. In October, the classification by Chl-*a* concentration considered all of points as hypereutrophic.

The classification using Chl-*a* obtained similar results with the TSI of the three CETESB monitoring spots inserted into the BBHR [43], with predominantly supertrophic and hypertrophic environments. In inland waters, the classification using Secchi disk must be used with caution because the transparency was associated not only with algal particles but also with inorganic particles [76,77,78].

The inclusion of the cirrus band improved the atmospheric correction results, eliminating negative values, mainly in the blue region. Typically, the atmospheric correction of Landsat images using FLAASH results in negative values for dark targets, such as water bodies. Negative values are associated with the overestimation of aerosol reflectance [62,63,64] and harm the performance of models that use blue bands [79]. Validation of the atmospheric correction showed that there was a difference in the magnitude between the simulated *Rrs* and OLI imagery *Rrs*. Likely, all of the influence of the glint effect or aerosol was not removed. Differences in the magnitude were eliminated when bands algorithms were used. On the other hand, differences in the magnitude negatively influenced the models that used only one band. 

## 6. Conclusions

Overall, satisfactory fits were obtained from simulated OLI/Landsat-8 bands. The NIR-Red, NIR-Green and NIR-Blue ratios yielded an R^2^ greater than 0.70. These results showed that OLI bands were sensitive enough to detect Chl-*a* concentration. Nevertheless, validation of the models showed that fitted models did not have accurate results for estimating the Chl-*a* concentration. The NIR-Red ratio yielded the best fits, both linear and polynomial. The NIR-Green ratio obtained minor errors and did not achieve satisfactory results.

All of the fitted models showed problems of overestimation, except the polynomial NIR-Green model that underestimated the Chl-*a* concentration. Validation showed that polynomial NIR-Green model underestimated high concentrations; however, it also overestimated low concentration that impaired the classification of the trophic state. The fitted empirical models had estimated values greater than the threshold for hypereutrophic water (69.05 mg·m^−3^). Hence, the products of Chl-*a* supplied for these models were unsuitable for low trophic environments. The models were not able to include the range of variations in the Chl-*a* concentration observed in the BBHR. Consequently, maps of the trophic levels yielded from the Chl-*a* concentration estimated by these models would not present the reliable variation that can be seen in the representation by points.

Although the fitted models did not yield accurate estimates of the Chl-*a* concentration, due the good sensitivity of the OLI/Landsat-8 with Chl-*a*, it is still possible that OLI/Landsat-8 bands are suitable for other aquatic systems with characteristics different than the BBHR.

Among the bands-based algorithms proposed by the literature and tested, the 3B models obtained satisfactory results when it they were tuned using the field data. Validation results were greater than the fitted models using simulated OLI bands. Unfortunately, we did not have an image of the study area with bands compatible for applying the model and having a spatial representation Chl-*a* concentration of the whole reservoir. However, the models would likely yield satisfactory spatial representations of the Chl-*a* concentration variations along the BBHR.

Although the models were calibrated from MERIS sensor (not operational) wavelengths, satellite data of sensors with equivalent wavelengths can be adopted, such as MODIS, Sentinel-2 (both operational), and Sentinel-3 (the first satellite is scheduled for launch in the fourth quarter of 2015). Bands of these sensors likely have suitable sensitivity for estimating the Chl-*a* concentration and, consequently, the trophic state. Furthermore, these sensors present a band at approximately 620 nm, allowing the estimation of the phycocyanin pigment. Thus, periodic monitoring can be accomplished and alerts could be issued for the population.

The calibrations proposed in the literature did not obtain satisfactory results. The calibration proposed by [72] for 3B combination had similar results when compared to the best results obtained by the fitted models. The unsatisfactory performance of these calibrations showed that was not possible to obtain a global empirical model (suitable to other time periods and regions), even when using a large calibration dataset.

Finally, more studies must be completed to improve the models in the BBHR. The inherent optical properties must be investigated better and applied to other approaches, such as semi-analytical and quasi-analytical models for estimating the Chl-*a* concentration.
